# Communications Skills in the Pharmacy Profession: A Cross Sectional Survey of UK Registered Pharmacists and Pharmacy Educators

**DOI:** 10.3390/pharmacy6040132

**Published:** 2018-12-12

**Authors:** Zahraa Jalal, Anthony Cox, Neera Goel, Nikita Vaitha, Kathryn King, Jon Ward

**Affiliations:** 1 School of Pharmacy, University of Birmingham, Birmingham B15 2TT, UK; a.r.cox@bham.ac.uk (A.C.); neera_goel@live.co.uk (N.G.); nikitavaitha@hotmail.co.uk (N.V.); j.d.ward@bham.ac.uk (J.W.); 2 Florence Nightingale Faculty of Nursing, Midwifery & Palliative Care, King’s College London, London SE1 8WA, UK; kathryn.king@kcl.ac.uk

**Keywords:** consultation skills, communication skills, motivational interviewing, pharmacists, pharmacy undergraduate training

## Abstract

**Objectives:** To determine UK pharmacists’ experiences of their current communication skills and undergraduate training and to identify communication skills training and teaching at UK schools of pharmacy. **Methods:** Two surveys were developed. The first survey was sent to UK practicing pharmacists examining their current communication skills and interest in behavioural counselling techniques such as Motivational Interviewing (MI). A second survey was sent to all UK Schools of Pharmacy investigating communication skills training and teaching. **Results:** In the first survey pharmacists reported low satisfaction with their undergraduate communication skills training. A convenience sample of 109 UK pharmacists responded to the first survey. Forty-four per cent (n = 48) of the respondents stated that they continued their professional development in communication skills after an undergraduate degree. Seventy (65.4%) were not familiar with behavioural counselling techniques such as MI. The most common patient consultation delivered by pharmacists was around adherence to medicine 22.4% (n = 50). Pharmacists expressed a need for further training in clinical areas such as mental health 25.7% (n = 80). Results from the second survey to pharmacy schools showed that Schools of Pharmacy response rate was 60% (18/30). All 18 schools stated that they teach health behaviour change consultation skills and this is mostly delivered by a clinical pharmacist. Teaching communication skills was mostly delivered as role play with peers (n = 17). **Conclusion:** This first national survey of communication skills training in Schools of Pharmacy shows that newer graduates have received more communication training compared to older graduates, however pharmacists’ respondents still felt that they were under prepared for behaviour change patient consultations. MI training would be welcomed by those. **Practice Implications:** Structured courses in communication skills, including behavioural change techniques, are needed for practicing UK pharmacists.

## 1. Introduction

Effective communication between healthcare professionals and patients is essential [1]. Evidence has shown that there is a strong positive relationship between a healthcare professional’s communication skills and a patient’s capacity to follow through with medical recommendations [2]. Communication skills are the basis for a more positive patient–clinician relationship which can lead to greater patient satisfaction and better outcomes [3]. Promoting health and wellbeing, in addition to disease prevention and medication management are considered current core services of pharmacy practice. Effective communication skills for pharmacists are important to cope with their evolving and expanding roles. Pharmacy professional bodies promote good communication between pharmacists and patients [1]. Regulators, such as the UK’s General Pharmaceutical Council (GPhC), set standards for pharmacists and pharmacy technicians which emphasise communication skills in ‘person-centred’ consultations [4]. Person-centred care, building a partnership between the patient and healthcare professional, provides a collaborative approach which can increase the likelihood of healthy behavioural change [5,6]. This changing professional relationship which is based on trust and mutual decision-making has created challenges for pharmacists [6]. As traditional models of medication provision are superseded, pharmacists have to develop innovative communication and consultation strategies, adapt new techniques, and reconfigure their service provision [6].

In the UK, the Centre for Pharmacy Postgraduate Education (CPPE) offers continuing professional development opportunities (CPD) and learning materials to support pharmacy professionals to develop and improve their consultation skills [7]. Pharmacy educational institutions in the UK offer an undergraduate Master of Pharmacy (MPharm) degree which is currently accredited by the GPhC. On completion of the MPharm degree, students undertake a year of preregistration training in an approved hospital or community pharmacy, before they can register as a pharmacist. Currently some UK Schools of Pharmacy also offer a preregistration integrated five year programme. Internationally, The International Pharmaceutical Students’ Federation (IPSF) and the International Pharmaceutical Federation (FIP) have promoted patient consultation skills since the 1980s. They currently publish educational tools and booklets to enhance communication skills and learning techniques for pharmacists. A number of their publications, in multiple languages, have targeted pharmacy students, pharmacy schools, and professional associations [8]. Concordance, the mutual agreement between the patient and healthcare professional, was a key element of these publications [6].

Methods of achieving such patient focused consultations behavioural strategies include health coaching (HC) and motivational interviewing (MI) that can be employed as consultation styles and approaches. Although, both can be effectively used to achieve behaviour change in healthcare, and although techniques could overlap, both remain distinct [9]. MI is a client-centred, directive style of counselling developed by Miller in 1983, from his experience in the treatment of problem drinkers [10]. It consists of four key principles of expressing empathy, developing discrepancy, rolling with resistance, and supporting self-efficacy [11,12,13]. MI is based on three key elements: collaboration, evoking or “drawing” out the client’s ideas about change, and emphasizing the autonomy of the client. In MI the interviewer should express empathy through reflective listening and develop discrepancy between clients’ goals or values and their current behaviour. Argument and direct confrontation should be avoided. The interviewer should also adjust to a client resistance rather than opposing it directly and support both self-efficacy and optimism [11,13]. MI employs Open Ended Questions, Affirmations, Reflections, and Summaries (OARS), often called microcounselling skills. MI also uses a simple tool for determining clients’ readiness to change, called the readiness ruler [14]. Patients are asked to rate on a scale of 1 to 10 how important a change is and the confidence they have in achieving that change. The effectiveness of MI for improving multiple chronic diseases and health issues, often focused on medication adherence in pharmacy consultations, has been demonstrated through evidence-based studies over the past decades [15,16,17,18,19,20].

Most recently Health Coaching (HC) has emerged in the last decade as another style of effective communication between patients and healthcare providers and has been rapidly operationalised across numerous healthcare sectors [9,10]. Numerous HC models and associated tools exist to support healthcare professionals in their practice. A number of validated approaches exist: the GROW model (Goals, Reality, Options, and Way forward), the four Es (Explore, Educate, Empower, and Enable), and the five As Approach (Ask, Advise, Assess, Assist, and Arrange) [5].

Unfortunately, there is little understanding of registered pharmacists’ perceptions of their current communication skills, and to what extent behavioural change approaches are integrated into UK undergraduate pharmacy curriculums [10]. Internationally, some pharmacy students are exposed to the concept of effective communication within weeks of beginning their formal education, whereas others do not receive patient communication training until after graduation [6]. Teaching principles of behaviour modification to pharmacists early at undergraduate level can allow time for pharmacists to develop and maintain competencies needed in new approaches to the medicine user [6]. Changes in UK pharmacy educational standards have focused on the increased clinical aspect of pharmacy practice, including communications since 2011, with outcomes such as “Establish and maintain patient relationships while identifying patients’ desired health outcomes and priorities” [21] but it is not known how this has influenced practice. This study seeks to discover the nature of communication training in Schools of Pharmacy, and how current registered pharmacists judge their own communication skills training experience.

### 1.1. Research Questions

(1) What is the self-assessment of UK practicing pharmacists regarding their consultation skills and their views on behavioural change consultations?
(2) What is the prevalence and nature of communication training and behavioural change consultation approaches in UK undergraduate pharmacy curricula?

### 1.2. Aims and Objectives

To determine UK pharmacists’ experiences of their current patient consultation skills and their perception of communication skills training at undergraduate level.

To determine current communication training in UK pharmacy education and describe the structured learning activities within them.

To explore if behaviour change consultation approaches, such as MI, are currently taught at UK pharmacy schools and are familiar to UK practicing pharmacists.

## 2. Materials and Methods

### 2.1. Survey to UK Pharmacists

An online survey, hosted on Bristol Online Survey, was distributed via an email or via an identical paper-based questionnaire to practicing pharmacists within the East Midlands, West Midlands, and London area by convenience sampling (the sample was collected by convenience sampling, that is to say data collection from pharmacists with established links with the university who were conveniently available to participate in the study). As emails were distributed to community pharmacy email addresses the number of individual pharmacists who could have responded to the survey cannot be confirmed, and therefore, a response rate could not be derived. Reminders were sent out via email in order to increase the response rate. The survey to practicing pharmacists was to determine pharmacists’ general views on their current consultation skills, training received at both undergraduate and postgraduate level on communication skills and to investigate whether there is an interest among practicing pharmacists to incorporate behavioural approaches, such as MI, into their pharmacy consultations. Before answering specific questions about MI, respondents were asked to view a short video which demonstrated the difference between a traditional consultation and a consultation using a MI approach.

Piloting of the survey questionnaires for the practicing pharmacists was done with a convenience sample of two pharmacists before distribution; these results were not included in the final sample.

### 2.2. Survey to Schools of Pharmacy

An online survey, hosted on Bristol Online Surveys, was distributed via an email link to 30 Schools of Pharmacy across the UK. The survey was sent by email to the MPharm programme directors, asking them to distribute the survey to the relevant person responsible for teaching communication skills at their schools. The survey to the Schools of Pharmacy investigated the breadth and delivery of components which are currently taught on clinical communication courses within curricula of UK Schools of Pharmacy and also aimed to explore whether behavioural change approaches were included in these courses.

Ethical approval: The research project was granted ethical approval from the School of Pharmacy Ethics Committee, at University of Birmingham. Responses received were anonymous and no individual information which could lead to respondent identification was required.

Statistical analysis: The results of the questionnaires were processed and analysed using statistical analysis software Statistical Package for Social Sciences programme (SPSS) version 23 (IBM Corp., Armonk, NY, USA).

## 3. Results

### 3.1. Survey To Practicing Pharmacists

A convenience sample of 109 registered pharmacists responded to the survey, with 42% (n = 45) having more than 10 years experience working as a pharmacist and 60% (n = 67) of the respondents practising as community pharmacists (see Table 1). There was approximately an equal gender split in the respondents.

#### 3.1.1. Pharmacists’ Consultation Skills Taught at Undergraduate Level

Half of the pharmacist respondents (51%, n = 54) stated they had not received teaching on consultation skills at undergraduate level, with a further 2% not sure. From the 42% (n = 45) of pharmacists who had more than 10 years’ experience working as a pharmacist only 11% (n = 5) were taught consultation skills at undergraduate level. Of the 32% (n = 34) of pharmacists who qualified in the last five years all except two were taught consultation skills at pharmacy school.

Of the 47% who were taught consultation skills during their undergraduate education, the most common delivery of consultation skills teaching was via small group teaching sessions (23.4%, n = 32), followed by lectures/forums (22.6%, n = 31). A small proportion of respondents (14.6%, n = 20) were taught via role play with simulated patients. The least common (8%, n = 11) approach respondents received teaching of consultation skills was via problem based learning.

Participants were asked to rank how valuable the teaching they received at undergraduate level was in developing their consultation skills on a scale from 1 to 10, with 1 being not valuable at all and 10 being very valuable. Of the 47% of pharmacist respondents who had been taught consultation skills at undergraduate level, more than half (56.5%, n = 30) reported that they were not satisfied with this training; ranking their consultation skill training as below 5.

#### 3.1.2. Pharmacists’ Consultation Skills Taught at Postgraduate Level/Continuous Professional Development

Forty eight (44%) of the pharmacist respondents stated that they had continued their professional development in communication skills. This was achieved by online courses (20.1%, n = 40), self-reading (19.6%, n = 39), and from attending workshops (face-to-face training sessions) (10.1%, n = 20). Few pharmacists (1.5%, n = 3) developed their consultation skills during their postgraduate degree. The degrees included a Doctor of Philosophy (PhD), Independent Prescribing course (IP), and a MSc. in Advanced Clinical Practice. A small proportion (11.1%, n = 22) of pharmacist respondents had not carried out any postgraduate courses or continuous professional development to further develop their consultation skills.

#### 3.1.3. Training on Consultation and Communication Skills

**Figure 1:**
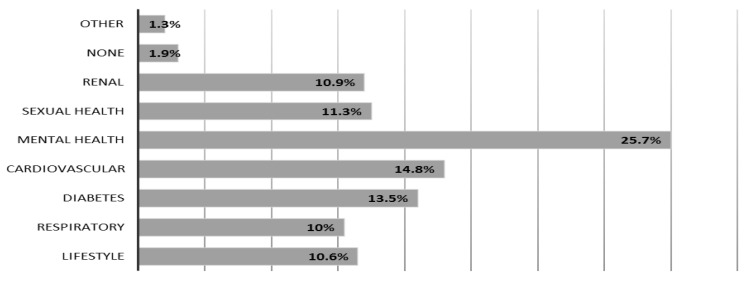
Clinical areas that pharmacists reported they require further training. Note: Participants were allowed to select more than one option; the number of options selected was 311 by 109 participants.

The majority of the pharmacists (90%, n = 94) believed that they needed further training on their consultation skills and only (10%, n = 11%) did not agree. There was a range of clinical areas where pharmacists reported that they needed further training, especially to allow them to approach patients. These included mental health (25.7%, n = 80), cardiovascular (14.8%, n = 46%), diabetes (13.5%, n = 42) sexual health (11.3%, n = 35), renal diseases (10.9%, n = 34), and respiratory (10%, n = 31) as shown in Figure 1 below.

Most (67%, n = 72) of the practicing pharmacist respondents reported that they would be interested in receiving MI training after watching the videos provided in the questionnaire link. Fifteen percent of respondents (n = 16) stated that they would not be interested in training on MI, whilst a small proportion (18%, n = 19) responded with ‘maybe’.

#### 3.1.4. Pharmacists’ Knowledge and Application of Behavioural Interventions

Regarding pharmacists’ awareness and knowledge of MI as a consultation approach, the results showed that the majority (65.4%, n = 70) were not very familiar with the term MI. A very small proportion of pharmacist respondents (3.7%, n = 4) had received formal training on MI interviewing at undergraduate level, and few participants received training at postgraduate level (9%, n = 10) and from external workshops (10%, n = 11).

**Figure 2:**
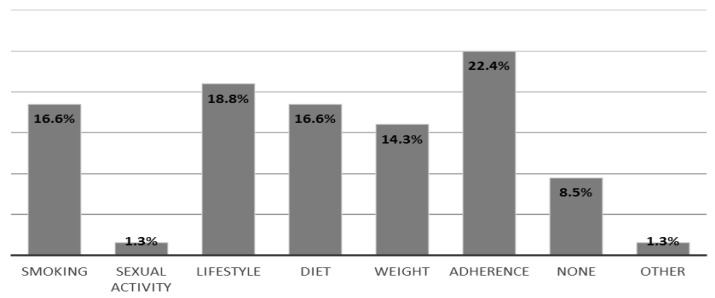
Behavioural changes pharmacists observed in their patients after carrying out pharmacy consultations. Note: Participants were allowed to select more than one option; the number of options selected was 223 by 109 participants.

Pharmacist respondents were asked if they had observed behavioural changes which they felt had occurred due to any advice they had given to their patients (Figure 1). The most common (22.4%, n = 50) response was a positive change in adherence to prescribed medication. Around (18.8%, n = 42) of pharmacist respondents stated that they had observed changes in patients’ lifestyles after carrying out consultations. This included smoking cessation (16.6%, n = 37) and healthy diet change (16.6%, n = 37). Weight reduction was observed less frequently (14.3%, n = 32). Other behavioural changes included (1.3%, n = 3) alterations in healthy sexual activity and improved self-care management (1.3%, n = 3). A small proportion (8.5%, n = 19) of pharmacists’ responded that they had not observed any behavioural changes in patients after carrying out their consultations. The remaining pharmacists reported that they were never given the opportunity to follow-up patients after a patient consultation, and therefore had not observed any behavioural changes in their patients and were not able to provide such information (please see Figure 2).

### 3.2. Survey to Schools of Pharmacy

The survey was sent to 30 UK Schools of Pharmacy and 18 responded giving a response rate of 60%. Further data on MPharm variations and cohort sizes can be found in Table 2.

#### 3.2.1. Methods of Teaching Communication and Consultation Skills

The method of teaching clinical communication skills among the 18 pharmacy schools was mostly by role play with peers (94%, n = 17) and Small Group Teaching (88%, n = 16). A large number of schools involved real patients in teaching (observed consultations with real patients) (83%, n = 15) and video/digital recording of student consultations also took place in teaching sessions (83%, n = 15). Other methods of teaching included role play with simulated patients (77%, n = 14), role play with academics (72%, n = 13), video consultation exemplars (66%, n = 12), Problem Based Learning (38%, n = 7), and Forums (22%, n = 4). One school stated that they include role play and forum theatre with actors in their teaching, another school taught Team Based Learning activities, and a third school stated that they relied on placements for the students to learn consultation skills.

#### 3.2.2. Health Behaviour Change Teaching

All 18 schools who responded to the survey stated that they teach some form of health behaviour change in their communication skills teaching curriculum and again this was also mostly taught as role play with peers followed by small group teaching and lectures.

#### 3.2.3. Teaching of Communication/Consultation Skills

Sixteen schools (88%) replied that a clinical pharmacist is the main responsible person for development and delivery of communication skills including behaviour change counselling on their MPharm programme. Communication skills behaviour change is taught by an educational specialist at four schools (22%), a clinical psychologist at (16%, n = 3) schools. In one school (5%) a health psychologist provided communication teaching and two schools (11%, n = 2) employ a specialist clinical communication tutor. Two schools (11%) employ a community based medicine general practitioner for teaching communication skills.

#### 3.2.4. MI in an MPharm Curriculum

Twelve schools (66%) stated in the survey that they teach motivational interviewing as part of their curriculum and in eight (44%) schools this was taught and delivered by a clinical pharmacist. To ensure that the schools are incorporating the main elements of MI, they were asked to list any acronyms they used that are associated with MI, as reported by the schools these included DARN-CAT [22], EARS [23], OARS [24], PACE [25], and REACH [26] (see Table 3). In the practical part of teaching MI, eleven schools (61%) specified that they used simulated patients/actors. All schools (n = 18) agreed MI should be important to develop for their pharmacy communication teaching, with twelve already using MI to some extent.

#### 3.2.5. Collaboration with Other Schools/Departments

Strategic collaboration with other departments/schools was with medicine (38%, n = 7), nursing (22%, n = 4), sociology (16%, n = 3), and psychology (16%, n = 3). Psychotherapy and counselling collaboration occurred in (5%, n = 1) pharmacy school.

#### 3.2.6. Assessment of Motivational Interviewing

Four out of 12 schools who teach MI on their course stated that they do not assess MI skills during their programme. The remaining eight schools who assess MI do so across the 4–5 years, with assessments mostly taking place in year 3 of the MPharm degree (n = 7). Six schools stated that they use formative assessments to assess their teaching on MI and five stated that their assessment is summative.

## 4. Discussion

This is the first national study examining current communication skills training in UK undergraduate pharmacy education and perspectives of registered pharmacists on their current consultation skills since new educational standards in pharmacy were implemented. Key findings show that all schools, who responded to the survey, incorporate health behaviour change in their communication skills curriculum. Delivery of patient consultation skills is mostly as role play with peers followed by small group teaching and lectures. Role play is useful in that it allows practicing communication in a safe and controlled environment [27]. It also gives a space for learners to practise and receive feedback [27]. Previous studies have shown that role play is effective in enhancing communication skills [28,29], in addition recent studies have shown that students consider peer-role play as an essential tool to acquire effective communication skills [30].

However, there are limited comparative studies examining the effectiveness of peer role play and simulated/standardised patient role play available in the literature. In previous studies, one study [31] identified a potentially unhelpful lack of realism associated with peer role play. Another study [32] recognised the potential for standardised patients to provide professional feedback which could benefit the training of specific communication skills and a further study [33] concluded “Assessment by clinical supervisors indicates that communication training modules including standardized patients and an Objective Structured Clinical Examination “OSCE” are superior to communication training modules with peer role playing”. The positive outcomes possible from the inclusion of simulated/standardised patient role play in undergraduate pharmacy education have to be balanced with attendant pressures regarding financial and faculty implementation efforts.

Clinical pharmacists without formal communication education (i.e., a university education in communication science) appear to be the main academic group delivering consultation skills at the pharmacy schools, although a few schools also employed psychologists and specialist communication tutors. Several questions could be raised; should current practice of teaching clinical communication skills be delivered separately from clinical skills? Do all schools need to employ specialists in communication skills to deliver such training? How equipped do academic clinical pharmacists feel to be able to deliver effective communication skills? Currently we could not find any consensus view regarding the qualities that are important for healthcare academic professionals to possess if they are going to be teaching clinical communication. There are studies that have investigated characteristics of medical and clinical educators in general not specific to communication skills and what makes an exceptional clinical teacher and show that being enthusiastic and having clinical competence are important attributes. Personal attitudes, communication skills, educational knowledge, and educational skills are all thought to be important [34,35]. Regarding behaviour change teaching there is no current evidence showing that any one type of health professional is inherently better than any other [36].

A high proportion of schools reported behavioural change strategies such as MI at UK pharmacy schools were being taught (12/18, 66%). Schools currently include MI in their curricula, with the desire of those schools who do not currently teach these skills to adopt these methods. A similar finding was found in a study on pharmacy education in the United States (US), results of a survey of faculty members teaching communication skills in schools of pharmacy across the US found that around 65% of the survey respondents reported covering MI concepts in their communication course [37]. MI is currently recommended as routine training for US speciality pharmacists [38]. While a survey from Europe including 11 Nordic pharmacy schools showed that training in patient communication skills is still limited and that there is a need to restructure communication skills training to help future pharmacists provide effective patient care [39]. As a majority of Schools of Pharmacy include MI in their curricula, further research needs to investigate how MI skills are assessed at pharmacy schools. This can be achieved by using specific validated tools designed to assess MI fidelity and provide feedback on the acquired skills.

Our results provide some evidence that education at UK pharmacy schools may have shifted from a biomedical, paternalistic model of communication which was predominant through the 1900s [40], to more ‘patient-centred’ model, reflecting wider changes in medical, and other healthcare professional groups, curricula. More specifically patient-centred models for communication during consultations (both behaviour and task focused), including models or frameworks based on the work of Balint (1957), Berne (1964), Heron (1976), Byrne and Long (1976), Pendleton et al. (1984), Neighbour (1987), and Kurtz & Silverman (1996) among others) [41] during the latter half of the twentieth century evidenced a significant change in the understanding of doctor–patient interactions and subsequently provided motivation for changes within undergraduate healthcare professionals’ education. This change is driven by policy and regulatory bodies such as the Department of Health with a call for “empowerment of patients in their own healthcare decisions” and also in the Patient’s Charter 1991 [42]. Regulatory bodies reflected the move towards patient or partnership-focused interactions within key documents, such as Tomorrow’s Doctors (GMC, 1993, 2003, 2009), Promoting Excellence 2016, and Good Medical Practice (GMC, 2014) [43,44,45,46,47]. This shift is also supported by The Royal Pharmaceutical Society’s (RPS) ‘Now or Never’ report [48], and the RPS’s ‘Medicines optimisation guidance’ which highlight the patient experience and self-management [49].

The current GPhC guidance on undergraduate pharmacist education published in 2011 emphasise the provision of person-centred care and tailored communication to improve partnership working with patients [21]. This may explain why the vast majority of recently qualified pharmacists in our study report having undergraduate communication training. However, it does not suggest specific frameworks for teaching and assessing communication skills. While it may seem desirable for a regulator to consider specifying particular frameworks, such as MI, it creates strictures which may prevent innovation by educationalists being guided by new pedagogic research evidence.

However, this study also shows that around half of the registered pharmacists had not received consultation skills teaching at undergraduate level, with half of this subgroup completing pharmacy school education more than a decade ago. There remains a need for increased provision and uptake of communication training at the postgraduate level, to ensure pharmacists can deliver on new roles involving increased need for consultation skills. There are opportunities available for registered UK pharmacists to improve and develop their consultation skills. These include online courses provided by CPPE and Health Education England. In addition, to face to face commissioned courses on behavioural approaches such as MI and HC exist. These training courses need to be endorsed, uniform, delivered in a structured manner and made available to all UK registered pharmacists.

A 2012 national UK survey of 700 pharmacist respondents found approximately 30–40% of pharmacists felt not fully prepared by undergraduate consultation skill training [50], and this matches our findings in less recently qualified pharmacists. While more recently qualified pharmacists acknowledge a greater degree of undergraduate training in consultation skills, significant numbers of registered pharmacists seek training at postgraduate level, mostly through self-development. The majority of pharmacist respondents were not very familiar with MI and the uptake and use of such techniques by UK pharmacists appears limited. Studies have shown in the field of medication adherence [20,51,52] that MI interventions can be both effectively delivered by pharmacists and can be effective in improving adherence in cardiovascular and other chronic disease populations. However, the evidence is still limited and there is a need for multicentred randomised controlled trials that could provide more conclusive evidence regarding the impact of MI on patients’ clinical outcomes.

Evidence of adoption of comprehensive communication teaching within Schools of Pharmacy is limited with only five published, peer-reviewed articles from the UK relating to communication training in pharmacy education identified in a review of the literature between 1995 and 2010 [53]. Our survey provides updated evidence on current pharmacy communication skills education from UK pharmacy schools, suggesting communication training at undergraduate level is now widespread.

The majority of pharmacists included in this survey expressed interest in training on behaviour change strategies including MI, and a quarter of respondents indicated that mental health is an area where they felt they would benefit from further training. MI was originally developed for the treatment of substance abuse but has rapidly expanded to other major mental health populations beyond addictions [54]. This is in line with the current NHS focus on mental health as a priority, with a drive towards an equal response to both mental and physical health [55].

Limitations to this study include the small sample size of the practicing pharmacists’ survey and response rate of pharmacy schools, although the majority of schools did reply. However, this study adds to the limited evidence on behavioural approaches in communication skills in UK pharmacy schools’ curricula.

### Practice Implications

There is a need to increase the availability of training courses for postgraduate registered UK pharmacists including behavioural change approaches; in addition, regular revalidation of recent pharmacy graduates on their communication skills and techniques would be important. Finally, there is a need to maintain and develop the pharmacy curricula for future undergraduate courses with behavioural change techniques such as MI and HC as integral components.

## 5. Conclusions

This first national survey of communication training in Schools of Pharmacy provides evidence that Schools of Pharmacy have embedded communication training, including MI, into curriculums, and there is some evidence that newer graduates have consultation training. However, a significant proportion of community pharmacists may be underprepared to conduct person-centred consultations aimed at influencing health behaviours in a way that would positively affect patient outcomes. Postgraduate training in MI techniques would be welcomed by those pharmacists.

## Figures and Tables

**Table 1:** Pharmacists’ demographics (N = 109).

Characteristics	N (%)
**Gender**	
Male	53 (50%)
Female	52 (49.1%)
Prefer not to say	1 (0.9%)
**Qualified pharmacist (years)**	
0–5	34 (32.1%)
6–10	27 (25.5%)
More than 10	45 (42.5%)
**Type of community pharmacy**	
Chain	40 (39.6%)
Independent	34 (33.75%)
Locum pharmacist	13 (12.9%)
N/A	14 (13.9%)
**Years of pharmacy practice (years)**	
31+	5 (4.7%)
25–30	11 (10.3%)
20–24	14 (13.1%)
15–19	8 (7.5%)
10–14	14 (13.1%)
5–9	21 (19.6%)
0–4	34 (31.8%)
**Branch of practice**	
Hospital	20 (18%)
GP surgery	8 (7.2%)
Community Pharmacy	67 (60.4%)
Other	16 (14.4%)
**Region of work**	
East Midlands	38 (35.5%)
West Midlands	47 (43.9%)
London	8 (7.5%)
Other	14 (13.1%)
**Qualification**	
MPharm	70 (57.9%)
BSc Pharm	40 (33.1%)
Pharma D	0
Independent Prescriber	11 (9.1%)

**Table 2:** Survey results from UK Schools of Pharmacy.

School of Pharmacy	MPharm Variations	Cohort Size (2016–2017)	In What Year Are Communication/Consultation Skills Included in Your Curriculum	Communication Course Includes Strategic Collaboration with Other Departments	Is Assessment for Behaviour Change Techniques (MI) Formative or Summative?
1	BOTH 4 Year and 5 Year MPharm	76–100	First Year, Second Year, Third Year, Fourth Year, Fifth Year	Medicine	Formative
2	4 Year MPharm	101–150	First Year, Second Year, Third Year, Fourth Year	Medicine	Not applicable
3	4 Year MPharm	51–75	First Year, Second Year, Third Year, Fourth Year	Medicine	Summative
4	4 Year MPharm	76–100	First Year, Second Year, Third Year, Fourth Year		Not applicable
5	4 Year MPharm	101–150	First Year, Second Year, Third Year, Fourth Year	Medicine, Nursing, Social work/sociology	Summative
6	4 Year MPharm	101–150	First Year, Second Year, Third Year, Fourth Year	None—no strategic collaborations	
7	BOTH 4 Year and 5 Year MPharm	101–150	First Year, Second Year, Third Year, Fourth Year, Fifth Year	Medicine, Nursing	Formative
8	4 Year MPharm	101–150	First Year, Second Year, Third Year, Fourth Year	Psychology	Not applicable
9	BOTH 4 Year and 5 Year MPharm	76–100	First Year, Second Year, Third Year, Fourth Year	Psychology, Psychotherapy and counselling, Medicine, Nursing, Social work/sociology	Formative
10	4 Year MPharm	76–100	First Year, Second Year, Third Year, Fourth Year	None—no strategic collaborations	Formative
11	4 Year MPharm	0–50	First Year, Second Year, Third Year, Fourth Year	None—no strategic collaborations	Summative
12	4 Year MPharm	101–150	First Year, Second Year, Third Year, Fourth Year	None—no strategic collaborations	Not applicable
13	BOTH 4 Year and 5 Year MPharm	101–150	First Year, Second Year, Third Year, Fourth Year, Fifth Year	None—no strategic collaborations	Summative
14	4 Year MPharm	76–100	First Year		Formative
15	4 Year MPharm	101–150	Second Year, Third Year	Psychology	Not applicable
16	4 year MPharm	0–50	First Year, Second Year, Third Year, Fourth Year	Medicine	Summative
17	4 Year MPharm	101–150	First Year, Second Year, Third Year, Fourth Year	Nursing, Social work/sociology	Not applicable
18	BOTH 4 Year and 5 Year MPharm	200+	First Year, Second Year, Third Year, Fourth Year, Fifth Year	None—no strategic collaborations	Formative

**Table 3:** Behavioural techniques acronyms.

ACRONYM	Elements of Behavioural Approaches
DARN-CAT [22]	Desire, Ability, Reason, Need—Commitment, Activation, Taking steps
EARS [23]	Elaborate, Affirm, Reflect, Summarise
OARS [24]	Open-ended questions, Affirmations, Reflections, Summaries
PACE [25]	Partnership, Autonomy/Acceptance, Compassion, Evocation
REACH [26]	Resist the ‘righting reflex’, Evoke, Affirm autonomy, Clarify goals, Highlight change talk
